# Circular from the Surgeon-General U. S. Army

**Published:** 1863-04

**Authors:** 


### CIRCULAR FROM THE SURGEON-GENERAL U. S. ARMY.

We take pleasure in calling attention to the following circular, which we take for granted is all that is necessary to secure the cordial and general co-operation of the profession in the collection of the information desired by the Surgeon-General. The facts thus accumulated cannot fail to contribute to the advancement of our science, and be most useful to the profession:

*Surgeon-General's Office,*
*Washington City, D. C., February* 20, 1863.

The Surgeon-General would remind the medical profession that, some months since, a medical officer was detailed by the department to prepare the surgical history of the rebellion. It is intended that this history shall embrace, among other topics, the collected results of the gun-shot injuries of the war, and of the operations performed for their relief.

Many facts, bearing on these subjects, can be obtained by an examination of the returns of the various military hospitals; and explicit orders have been issued to the surgeons in charge as to the manner of reporting. Yet it is found, practically, that the results of all cases cannot be included in these reports.

In every depot of wounded, and after every action, there exists a large class of injured men, who, in various stages of convalescence, pass from the observation and treatment of the military surgeon, and are lost sight of by the medical department. These patients are those who are either furloughed or discharged the service by military authority before their treatment is essentially terminated. Under such circumstances, all past records of these cases are rendered valueless from the absence of a positive knowledge of their results.

To remedy this evil the Surgeon-General appeals to the profession of the country, and solicits their co-operation. He would ask every physician and surgeon who may be called upon to treat any officer or soldier wounded in service, carefully to note the results of the case, to record his observations, and, when the case shall have terminated, to transmit a copy of his observations to the Surgeon-General's office.

The following form is suggested:—

*Form.* *Date of Communication.*

*Character of Injury.* *Name and address of Physician forwarding it.*

| | Where wounded and date. | To what hospital transported. | What operations, &c., performed. | By whom performed. | Date of furlough or discharge. | Present condition of patients. Acc't of case. Treatm't, &c. Result. |
|---|---|---|---|---|---|---|
| Patient's name and age. | | | | | | |
| " rank. | | | | | | |
| " Reg't & Co. | | | | | | |
| " postal address | | | | | | |

In all cases of recovery after *excisions* of bone, the amount and character of the movements executed by the patient, with the injured limb, should be accurately described. Where amputation has been practiced, the character of the stump should be noted, especially when the operation has been performed through an articulation. In cases of compound fracture the point of fracture should be stated, as also the degree of efficiency of the limb remaining after treatment. In compound fracture of the femur the amount of shortening should be measured, and the strength and usefulness of the limb described. In those patients in whom injuries of the skull have occurred, or upon whom the trephine has been applied, the mental and physical conditions should alike be dwelt upon.

In thus placing before the profession the objects he desires to obtain, the Surgeon-General trusts that he will meet with active co-operation. By the means above indicated much information that is valuable may be collected, and the interests of the science of surgery materially advanced.

W. A. HAMMOND, Surgeon-Gen'l U. S. A.

Medical journals will please copy.

